# A review of the clinical characteristics and management of immunosuppressed patients living with HIV or solid organ transplants infected with SARS-CoV-2 omicron variants

**DOI:** 10.3389/fpubh.2024.1327093

**Published:** 2024-02-22

**Authors:** Yan Song, Lixin Lou, Kaiyu Zhang

**Affiliations:** ^1^Department of Infectious Diseases, The First Hospital of Jilin University, Jilin, China; ^2^Department of Infectious Diseases and Center of Infectious Diseases and Pathogen Biology, The First Hospital of Jilin University, Changchun, China

**Keywords:** immunosuppression, HIV, organ transplant, SARS-CoV-2 infection, omicron, inflammation

## Abstract

The severe acute respiratory syndrome coronavirus 2 (SARS-CoV-2) omicron strain was first detected in South Africa in November 2021. Although clinical responses to SARS-CoV-2 depend on host immunity, it remains uncertain how immunosuppression affects subsequent coronavirus disease 2019-related (COVID-19-related) incidence, severity, and mortality, especially with respect to the omicron strain. Conversely, immunosuppressants are often thought to predispose to infection. To explore the associations between host immunity and infection with SARS-CoV-2 omicron variants, here we discuss two groups of immunosuppressed patients: organ transplant recipients, who generally receive exogenous immunosuppressants, and Human Immunodeficiency Virus (HIV)-infected patients, who often have disease-related immunosuppression. In summarizing the clinical features and prognoses of HIV-infected patients and human organ transplant recipients infected with SARS-CoV-2 omicron variants, we provide new insights into the pathogenesis of omicron SARS-CoV-2 and provide a framework for the management of these patients now and in the future.

## 1 Introduction

On November 9, 2021, severe acute respiratory syndrome coronavirus 2 (SARS-CoV-2) mutant B.1.1529 was detected for the first time in South Africa. On November 26, the World Health Organization (WHO) named it the “omicron” mutant strain and listed it as a variant of concern (VOC) (1). Compared with the wild-type strain, the omicron strain had over 30 mutations in the critical spike (S) protein sequence, of which 15 were in the receptor-binding domain (RBD). Omicron strains are divided into the main subtypes BA. 1, BA. 2, BA. 3, BA. 4, and BA. 5. While BA. 1 was initially the main subtype in many countries, the burden of BA. 4 and BA. 5 subtypes continues to increase globally due to their high infectivity and propensity for immune evasion, especially from vaccination and previous infections (2), because of its deletions and mutations that lead to increased transmissibility, higher virus-binding affinity, and antibody escape (3). However, the effects of many omicron mutations remain unknown, creating uncertainty about how the full combination of deletions and mutations affect virus behavior and susceptibility to natural and vaccine-mediated immunity (2).

Although omicron SARS-CoV-2 is less pathogenic than preceding strains and most patients have a good prognosis, the variant can still cause severe disease or death in vulnerable groups including the older adult, those with chronic diseases, pregnant and perinatal women, and patients with cancer. While clinical responses to SARS-CoV-2 depend on individual host immunity (4), whether immunosuppression is a risk factor for SARS-CoV-2-related incidence, severity, and mortality is still debated. On the one hand, immunosuppressed patients are generally thought to be at greater risk of serious infection (5). On the other, immunosuppression may serve to limit the disordered excessive inflammatory response caused by cytokine release syndrome (CRS) seen in some cases of severe COVID-19 (6), thereby favoring positive outcomes. Immunosuppression can be either disease-related, such as seen in acquired immune deficiency syndrome (AIDS) and blood malignancies, or iatrogenic, as seen in solid organ transplant recipients (SOTRs) receiving immunosuppression to prevent rejection or patients with cancer receiving chemotherapy (7, 8).

However, it is unclear how different SARS-CoV-2 strains affect the susceptibility, outcomes, and vaccination-related outcomes for immunosuppressed patients. To better understand the interactions between immunosuppression and SARS-CoV-2, especially with regard to more recent strains, here we review the clinical characteristics and prognosis of patients with HIV and solid organ transplants after infection with the SARS-CoV-2 omicron strain. In doing so, we discuss the possible underlying mechanisms to provide treatment guidance for COVID-19 in these vulnerable groups.

## 2 The clinical characteristics of the general population infected with omicron strains

Omicron is different to earlier strains in terms of pathogenicity and virulence. Most patients with omicron variants develop symptoms, mainly low-grade fever, cough, nasal congestion, mild headache, sore throat, and muscle soreness and, in some individuals, gastrointestinal symptoms such as diarrhea. Compared with the initial strains, few have smell/taste loss (9). Omicron persists in the community and continues to evolve. It appears to be over 70-times more infective of bronchial epithelial cells but only a tenth the infectiveness of alveolar cells than the delta strain (10), suggesting that while the omicron variant is highly infectious, it might cause less damage to lung tissue than the delta strain. This also helps to explain why patients infected with omicron exhibit more pronounced upper respiratory symptoms such as sore throat and cough. In terms of laboratory tests, patients with omicron SARS-CoV-2 have lower neutrophil counts, white blood cell counts, C-reactive protein (CRP), and SARS-CoV-2 Immunoglobulin G (IgG) antibody titers than patients with non-omicron SARS-CoV-2, while lymphocyte count, serum amyloid protein, erythrocyte sedimentation rate (ESR), interleukin 6 (IL-6), interleukin 8 (IL-8), tumor necrosis factor-α (TNF-α), tumor necrosis factor-γ (TNF-γ) are all higher (11), highlighting the systemic nature of the disease. Reports of clinical outcomes from omicron infection have varied. For example, IgG-positive patients infected with the omicron strain were shown to have shorter hospitalization times and those with fever longer hospitalization times (11), while others have shown that the hospitalization time of patients with the omicron variant was longer than that in patients infected with non-omicron virus (11). Nyberg et al. (12) reported that confirmed omicron cases had a 59% lower risk of hospital admission, a 44% lower risk of any hospital attendance, and a 69% lower risk of death than those with confirmed delta cases, and the risk reduction was age-dependent. Although RBD mutations in the omicron variant can significantly alter the binding of known antibodies and potentially allow vaccine escape (13), the presence of vaccine-related antibodies may still shorten the hospital stay and virus clearance times.

Omicron has also challenged protection from vaccines. Overall, T cell reactivity in post-vaccine specimens is largely preserved against omicron, indicating that vaccines utilizing the parental antigen continue to be protective against disease caused by the omicron variant (14). A systematic review supported booster-dose vaccine efficacy against SARS-CoV-2 variants (15). For people with normal immune function and no underlying diseases, even if breakthrough infection occurs, the symptom severity and the risk of hospitalization appear to be low and the recovery period short. Furthermore, in these individuals, the vaccine effect appears to persist. However, certain vulnerable groups, such as people living with HIV (PLWH) and SOTR, remain at higher risk of adverse outcomes from COVID-19, and continuing guidance on vaccination strategies and COVID-19 management is needed in these groups, especially to pre-empt any further changes in the virus.

## 3 Immunosuppression may be both harmful and beneficial in SARS-CoV-2 infection

Little is known about the course of omicron SARS-CoV-2 infection in immunocompromised patients, who are at presumed risk of more severe disease but who may also have decreased detrimental inflammatory responses. Due to impaired immune defenses from both underlying disease and treatment, immunocompromised patients with respiratory virus infections are generally at risk of more severe infection and increased rates of bacterial and fungal superinfection compared to their immunocompetent counterparts (16). However, the association between COVID-19 and cytokine release syndrome (CRS) (6) raises the possibility that immunosuppression may temper the exuberant inflammatory response in this unusual infection. Severe COVID-19 disease can cause CRS and secondary hemophagocytic lymphohistiocytosis due to innate immune activation, as also seen in patients with other viral infections (SARS-CoV, Middle East Respiratory Syndrome, Epstein–Barr virus) and patients receiving chimeric antigen receptor T (CAR-T) cell therapy (17). However, it remains uncertain exactly if and how different types of immunosuppression alter the clinical course of COVID-19 and, in the face of a dominant omicron strain, whether treatment and prevention measures initially developed for earlier strains need to be optimized to better manage these vulnerable patients.

## 4 The clinical characteristics and prognosis of people living with HIV infected with omicron strains

There are an estimated 38 million people living with HIV (PLWH) worldwide. PLWHs taking anti-retroviral therapy (ART) are generally not immunosuppressed but may be immunosuppressed if not adequately managed, the latter providing a human model of understanding SARS variant infection in the face of endogenous (disease-mediated) immunosuppression. Untreated HIV leads to decreases in CD4 lymphocytes in the blood and tissues associated with chronic inflammation (18). The United Nations AIDS Program lists HIV-positive patients as high-risk for COVID-19, and current thinking is that HIV infection may increase the risk of adverse consequences from COVID-19.

The reported incidence of omicron in PLWH varies. A recent study in China showed that COVID-19 is more common in PLWH than in the general population (19). Conversely, a prospective observational study of 29 PLWH and 114 healthcare workers analyzing immune responses after the third vaccination showed that after eliminating healthcare workers, the risk of omicron infection was close to the general population (20). When observed, the high incidence of COVID-19 in PLWH may be due to other associated comorbidities or social factors such as COPD, smoking, or drug or alcohol abuse (21).

In previous studies on other strains of the virus, the impact of HIV coinfection on the progression of COVID-19 may vary depending on the cohort population. In a small sample of SARS-CoV-2 single-infected individuals, HIV/SARS-CoV-2 co-infected individuals had a longer disease course (duration of viral nucleic acid positivity) and significantly delayed antibody reactions (22, 23). Geretti et al. (24) used the International Severe Acute Respiratory and Emerging Infections Consortium (ISARIC) database to compare and analyze data from 47,592 COVID-19 patients and adjusted for factors such as gender, age, and ethnicity. They showed that, compared to the general population, HIV-infected individuals with COVID-19 had a 69% increased risk of death. Consistent with the results of this study, Bhaskaran et al. (25) utilized the UK Open Safety platform to include 17,282,905 COVID-19 patients, including 27,480 HIV-infected individuals, and reported that HIV/SARS-CoV-2 co-infected individuals had a 159% increased risk of death compared with SARS-CoV-2 single-infected individuals. Regretfully, the kind of SARS-CoV-2 viral infection in patients has not been identified with precision in any of the aforementioned trials. Some studies have shown that the clinical manifestations of COVID-19 in PLWH are mainly mild, and the patients included in such reports are mainly people infected with Omicron. The aforementioned findings might be connected to Omicron’s pathogenicity given that its virulence and pathogenicity are now lower than they were previously (26). On the other hand, PLWH patients may have fewer breakthrough infections as a result of vaccination popularity. None of these studies provided information on the impact of HIV-related parameters (such as viral load, CD4^+^ T cell count, etc.) on hospitalization outcomes of COVID-19 patients. Tan et al.’s cross-sectional study (27) used an online self-completed questionnaire to assess the parameters associated with Omicron infection in PLWH and HIV negative individuals. Overall, the findings demonstrated that PLWH had a substantially lower rate of SARS-CoV-2 infection than did HIV-negative people. Nevertheless, not all asymptomatic carriers were tested for SARS-CoV-2 nucleic acid or fast antigen, and the study did not assess the clinical symptoms of the patients, which could lead to the missed identification of asymptomatic carriers. Further research is necessary to elucidate the characteristics of Omicron infection in patients with PLWH infection, as the asymptomatic carrier rate of Omicron virus is significantly greater than that of other VOCs (24).

In previous studies on other SARS-CoV-2 strains, the evidence shows that there are differences in variant behavior in patients: (i) with and without HIV; (ii) in PLWH with and without ART; (iii) in PLWH with high and low CD4 counts; and (iv) in PLWH and with and without vaccination (28). In a retrospective cohort study of 108,062 PLWH in the US in 2020, 2,988 were diagnosed with COVID-19. In this study, decreased CD4^+^ T cell counts were associated with increased risk of COVID-19 infection, hospitalization, and hospital death (29). Braunstein et al. (30) also found that the hospitalization rate of PLWH with CD4^+^ counts <200 cell/mL was higher than those with higher CD4^+^ counts and outcomes were poorer. CD4 lymphopenia is exacerbated by acute SARS-CoV-2 infection (31), and increases in inflammatory markers such as CRP and IL-6 are associated with a poor prognosis (28). A study conducted in South Africa grouping HIV-infected individuals based on HIV viral load and CD4^+^ T cell counts found that, regardless of the presence of viremia or immunosuppression, the mortality rate of COVID-19 patients with HIV positivity was significantly higher than that of HIV-negative patients, with viral load ≥1,000 copies/mL or CD4^+^ T cell counts <500/μL associated with the highest risk of death (32). Another study showed that the admission, mechanical ventilation, and mortality rates of HIV-positive COVID-19 patients in the intensive care unit showed an increasing trend compared to the general population, but the difference was not statistically significant (33). Similarly, Sigel et al. (34) found that there was no significant difference in the severity of and mortality from COVID-19 between HIV-infected individuals and HIV-negative individuals. These discrepant results may be due to differences in the study population; for instance, Karmen-Tuohy et al. (33) included HIV-infected individuals who received antiretroviral therapy before admission, and most of the patients had stable virus control and CD4^+^ T cell counts >200/μL.

However, active HIV replication or lower CD4+ T cell counts may be associated with more severe COVID-19 disease outcomes. Harter et al. (35) reported that two HIV/SARS-CoV-2 co-infected individuals who underwent antiviral treatment but did not achieve complete viral suppression developed critical illness, with one patient dying. Dandachi et al. (31) found that, compared with HIV-infected individuals with higher CD4+ T cell counts at baseline, for patients with CD4+ T cell counts less than 200/μL the risk of hospitalization and death from SARS-CoV-2 infection was higher. In addition, CD4+ T cells are essential for inducing humoral immune responses, and progressive CD4+ T cell depletion in HIV-infected individuals may hinder clearance of SARS-CoV-2 by affecting antibody production, leading to a prolonged disease course. Supporting this hypothesis, Israelow et al. (36) observed in a mouse model of acute SARS-CoV-2 infection that depletion of CD4+ T cells led to a weakened antibody response and delayed viral clearance. There is currently limited research on the outcomes of HIV infection with Omicron, but some research pointed out that cross-reactivity against Omicron generated by the vaccine appears to be mostly dependent on T cell response (37), and Omicron spike mutations occur in regions poorly targeted by CD4+ T cells (38). In all studied groups, the levels of CD4+ T cell responses to Omicron spike were consistently and significantly lower than those responsive to ancestral spike; this translated into a fold change that showed a median decrease in the CD4 response to Omicron of 14–30%. Considering the late stage of PLWH combined with CD4+ T cell depletion (39), based on existing research, we can speculate that effective antiretroviral treatment and COVID-19 vaccination for HIV-infected people are essential for reducing the SARS-CoV-2 infection rate, severity, and mortality in this population (Table 1).

**Table 1:** Summary of COVID-19 studies in PLWH patients.

Study	Number included	Major findings
COVID-19 outcomes among persons living with or without diagnosed HIV infection in New York State (29).	2,988 PWH with COVID-19	PLWH experienced poorer COVID-related outcomes relative to persons living without diagnosed HIV. With the decline in CD4^+^ T cell count, the risk of COVID-19 infection, hospitalization, and hospital death increased.
Coronavirus disease 2019 (COVID-19) infection among people with human immunodeficiency virus in New York City: a population-level analysis of linked surveillance data (30).	2,410 PWH with COVID-19	The hospitalization rate of PLWH with CD4^+^ counts <200 cell/mL was higher than those with higher CD4^+^ counts, and outcomes were poorer.
Characteristics, comorbidities, and outcomes in a multicenter registry of patients with human immunodeficiency virus and coronavirus disease 2019 (31).	286 PWH with COVID-19	Compared with HIV-infected individuals with higher CD4^+^ T cell counts at baseline, PLWH with CD4^+^ T cell counts <200/μL were at increased risk of hospitalization and death from COVID-19.
Clinical outcomes and immunologic characteristics of coronavirus disease 2019 in people with human immunodeficiency virus (28).	93 PWH with COVID-19	PLWH remain at risk of severe manifestations of COVID-19 despite ART, and those with increased markers of inflammation and immune dysregulation are at risk of worse outcomes.
Risk factors for coronavirus disease 2019 (COVID-19) death in a population cohort study from the Western Cape Province, South Africa (32).	3,460,932 patients (16% living with HIV)	Regardless of the presence of viremia or immunosuppression, the mortality rate of COVID-19 patients with HIV positivity was significantly higher than that of HIV-negative patients, with viral load ≥1,000 copies/mL or CD4^+^ T cell count <500/μL having the highest risk of death among HIV-infected individuals.
Outcomes among HIV-positive patients hospitalized With COVID-19 (33).	21 HIV-positive patients with 42 non-HIV patients using a greedy nearest-neighbor algorithm	HIV co-infection does not significantly impact presentation, hospital course, or outcomes of patients infected with SARS-CoV-2 when compared with matched non-HIV patients.
Coronavirus 2019 and people living with human immunodeficiency virus: outcomes for hospitalized patients in New York City (34).	88 PWH with COVID-19	There were no differences in adverse outcomes associated with HIV infection for hospitalized COVID-19 patients compared with a demographically similar patient group.
COVID-19 in people living with human immunodeficiency virus: a case series of 33 patients (35).	33 PLWH with COVID-19	SARS-CoV-2 infections may occur during boosted darunavir-based and/or on tenofovir-containing ART. HIV replication was active or associated with poor prognosis in COVID-19 patients.

The available data show that HIV-infected individuals who have received ART and whose CD4^+^ T cell count is within the healthy range have good tolerance to the COVID-19 vaccine, but some differences have been reported in terms of immune effect. Xu et al. (40) found that in their research on non-omicron strains, the seroconversion rate of HIV-infected individuals receiving two doses of the BNT162b2 vaccine was 98.7%, equivalent to the general population, but S protein-specific IgG levels were significantly lower than the general population. A phase II/III clinical trial conducted in the UK showed that the adenovirus vector vaccine ChAdOx1 nCoV-19 (AZD1222) induced effective humoral and cellular immune responses in HIV-infected individuals, which were maintained for at least 6 months, without any difference from the general population (41). Lombardi et al. (42) found that after receiving two doses of the mRNA-1273 vaccine, S protein-specific antibody levels and neutralizing antibody activity were similar in HIV-infected individuals and in the general population. Differences in age or sex composition, exclusion criteria (such as whether they had other underlying diseases), and the basic characteristics of HIV-infected individuals (such as HIV diagnosis time, viral load, CD4^+^ T cell count) could account for differences seen in various studies.

Research on omicron shows that, in addition to producing antibodies against vaccine strains, immunization with inactivated vaccines also produced neutralizing antibodies against D614G and delta mutant strains in most HIV-infected individuals, but the neutralizing antibody levels were significantly lower than those in the general population (43). Wang et al. (44) studied the humoral responses of individuals of different ages receiving different vaccination strategies infected with omicron BA.5/BF.7 before and after breakthrough infection in China from December 2022 to January 2023. They found that the newer variants showed increased immune evasion, and the effectiveness of prototype-based booster vaccine schemes on emerging variants such as CH.1.1 and XBB.1.5 continued to weaken. Repeated prototype-based booster vaccines may therefore not further enhance neutralizing antibodies against new mutations (44). Low levels of neutralizing antibodies are an important reason for SARS CoV-2 breakthrough infection (45), suggesting that antibody responses must be monitored in PLWH after COVID-19 vaccination. Compared to the general population, PLWH is more susceptible to breakthrough infection in the subgroup lacking a booster immunological dose, while Omicron is at much higher risk of breakthrough infection (46). Before and after the third dosage of the vaccine, El Moussaoui et al. (47) examined the relationship between vaccine-induced immune responses in PLWH and HCWs, including people who had not previously been infected or infected with SARS-CoV-2. The outcomes demonstrated a marked decrease in PLWH Omicrom-specific antibody responses. According to Alessandra Vergori et al.’s study (48), after receiving the third mRNA vaccine injection, all patients’ neutralizing activity against BA. 1 increased dramatically. However, independent of their HIV status, the neutralizing activity against BA. 1 was lower than that against the original W-D614G strain. According to Park et al.’s research, there was no discernible difference between PLWH and HCWs’ neutralization reactions on Omicron (43.94% vs. 51.77%, *p* = 0.42) (20). Well-managed PLWH and HCW exhibit a neutralizing response to the Omicron variation that is similar to that of HCW; nevertheless, the response in both species is significantly less than that of the wild type. As a result, even though PLWH have received three doses of the Omicron version that is now in circulation, there is still a substantial chance that this variety may lead to a breakthrough SARS-CoV-2 infection.

In conclusion, the information that is currently available suggests that the COVID-19 vaccination has less of a protective impact against Omicron mutant strains in HIV-positive individuals. It is vital to assess immune response in the ongoing COVID-19 and adjust the vaccination regimen as needed since HIV-positive individuals may have a lower immunological recall and durability to the SARS CoV-2 specific vaccine than healthy individuals. Furthermore, the majority of current research focuses on assessing the COVID-19 vaccination for adult PLWHs who have successfully controlled HIV replication through ART; less is known, however, concerning PLWHs in the juvenile age group or those who have not received ART or whose treatment outcomes are subpar. The information from these subgroups will give the immunization program for HIV-positive individuals a more thorough scientific foundation. The amount of matching evidence needed to justify the maintenance of the antibody protection efficacy is currently lacking. In order to provide more clarity, greater and larger scale study is still required in the future.

## 5 Clinical characteristics and prognosis of organ transplant recipients infected with omicron strains

SOTRs require iatrogenic immunosuppression, so they provide a human model of understanding SARS variant infection in the face of exogenous immunosuppression. It is generally believed that SOTRs infected with SARS-CoV-2 are at risk of more serious disease, complications, and a poor prognosis. For example, in one study, the incidence of pneumonia, the proportion of transfers to the ICU, and the mortality rate of SOTRs were significantly higher than in non-SOT COVID-19-infected patients (49). Among hospitalized kidney transplant recipients, the risks of secondary acute kidney injury and dyspnea were 3.78- and 4.53-times higher than for normal individuals, respectively, and these features indicated a poor prognosis (50). Kulkarni et al. (51) summarized 18 papers on COVID-19 infection in liver transplant recipients and found that the incidence of secondary acute renal injury after COVID-19 was 33.2%, the incidence of thrombosis was 5.8%, the incidence of bacterial infection was 11.6%, and the incidence of fungal infection was 2.6%. Bartlett et al. (52) found that age and potential complications were more important risk factors for severe COVID-19 in SOTRs than transplant-specific factors such as organ transplantation type, maintenance immunosuppression, and time after transplantation. Many reviews have suggested that in patients with COVID-19 infection after kidney transplantation, severe complications (dyspnea, acute renal injury, and acute myocardial infarction), severe disease, and fatality are significantly higher than in the general population (53–55). In a systematic review of 15 studies representing 223 liver transplant recipients with COVID-19 infection, the proportion of patients with severe disease (36%) or requiring hospitalization (19.3%) was very high, significantly higher than in the general population (56). Heart transplant recipients with COVID-19 infection showed similar demographic and clinical characteristics to the general population, but the prognosis was significantly worse (57). Furthermore, a number of meta-analyses on COVID-19 infection in SOTRs have shown that severity and mortality rates were significantly higher in these individuals than in the general population (57, 58).

SOTRs have a high risk of second infection with SARS-CoV-2. A study from the United States tracked 5,919 SOTRs between March 1, 2020 and March 30, 2021 and found that, in SOTRs, the incidence density of second infection with COVID-19 was 9.4 per 100,000 person days (59), much higher than that seen in a retrospective study on second infection in the general population (1.0 per 100,000 people) in Italy (60). In addition, immune evaluation of two SOTRs a few weeks before second infection showed that they had virus-specific CD4^+^ T cell responses and positive IgG titers before the second infection, which indicated that the risk of secondary infection was still high for SOTRs previously infected with COVID-19, even after generating cellular and humoral immune responses. There is currently no more research on the severity and mortality of SOTRs after secondary infection with COVID-19. The above studies have not identified the types of SARS-CoV-2 infections. Similar to the general population’s observed pattern, a sizable multicenter observational cohort study conducted during the Omicron period revealed that the death rate of SOTR hospitalized with COVID-19 dropped during the pandemic, even after adjusting for baseline comorbidities (61). In a different study (62), the incidence rate of COVID-19 induced by Omicron was still significant, but the fatality rate was very low among the highly vaccinated population of immunocompromised patients. Furthermore, 25% of patients experienced symptoms that persisted for longer than 30 days. This was significantly different from findings from a sizable community cohort of 29,000 UK citizens who had contracted Omicron (63). In this trial, 87% of participants—with a median age of 55—received the vaccination three times. This cohort and the Malahe et al. study (62) sample are similar in terms of age and immunization history. Only 1.9% of patients, however, need to be admitted to a hospital for treatment. Early COVID-19 medication may help recipients of kidney and lung transplants.

Allogeneic SOTRs must undergo iatrogenic immunosuppression to prevent immune rejection. Recipients take long-term immunosuppressive drugs, mainly calcineurin inhibitors (CNIs; usually tacrolimus or cyclosporine), as well as mycophenolic acid (MPA), mTOR inhibitors, and glucocorticoids. Maintaining the immune balance between anti-infection and anti-rejection is the key to improving the prognosis for SOTRs infected with SARS-CoV-2, but it is also a clinical challenge. Immunosuppressants can regulate several aspects of the host immune response, so the severity of COVID-19 may be influenced by the type, combination, and intensity of immunosuppression. For SOTRs with COVID-19, it is necessary to comprehensively evaluate graft function in this context and individualize treatment.

Many SOTRs infected with the omicron variant have now been vaccinated and treated with monoclonal antibodies, with promising outcomes of relatively low hospitalization and mortality rates (64). However, compared with the general population, SOT patients still experience higher hospitalization rates (65). Different studies have revealed differences in prognosis in SOT patients infected with omicron variants (64, 66), perhaps due to vaccine availability, newer COVID-19 therapies, and differences in the individual variants. However, there is currently no routine whole genome sequencing data to determine the precise omicron variants in this group of patients.

Mortality rates for hospitalized transplant recipients infected with omicron variants vary: 20% at a tertiary health center in Jeddah, Saudi Arabia (67), 4% in a US study (62), and 16% in Spain (65). Three doses of vaccine do not appear to reduce the mortality rate in transplant recipient patients (64), but early use of SARS-CoV-2 monoclonal antibodies (in addition to vaccination) is associated with preventing severe disease progression and oxygen demand (68).

There are currently insufficient data showing that omicron variants produce a different spectrum of disease. Abeer et al. (67) studied omicron patients (confirmed by whole genome sequencing) and did not find a specific omicron variant associated with poor prognosis. A recent study from South Africa showed that omicron B.A 1, 2, 4, and 5 were associated with decreased vaccine efficacy and an increase in hospitalization rates 4 months after booster vaccination. The authors suggested that patients should receive booster vaccines or new vaccines with protection against omicron variants 4 months after immunization to reduce COVID-19 hospitalization rates (69).

Given the different therapeutic effects of monoclonal antibodies on different variants, the use of monoclonal antibodies in transplant recipients during various SARS-CoV-2 waves has been challenging (70). In patients with compromised immune function, Evusheld, which included the clinical mAb combination of COV2-2196 and COV2-2130 (71), is recommended as a preventative prior to COVID-19 exposure, and its use was related to a reduction in the incidence of alpha, beta, and delta variants. Subsequent *in vitro* studies have shown that in the presence of SARS-CoV-2 variants BA. 4 and BA. 5, Evusheld offers poor protection against COVID-19, and it is ineffective against the new SARS-CoV-2 variants BQ and XBB. When these variants dominate in the community, their use should be carefully evaluated (71).

It is essential to understand whether SOTRs experience rejection and infection after receiving inactivated SARS-CoV-2 vaccine. At present, it is generally believed that the incidence of adverse reactions after receiving mRNA vaccines is similar in kidney transplant recipients to general results from clinical trials, and there are no safety issues (72, 73). After receiving the SARS-CoV-2 mRNA vaccine, the humoral immune response of kidney transplant recipients was lower than that of the general healthy population (74). However, through testing SARS-CoV-2-specific T cells, it was found that the cellular immune response of kidney transplant recipients receiving mRNA vaccine may be superior to the humoral immune response (75). Another study showed that kidney transplant recipients previously infected with SARS-CoV-2 can experience a very strong humoral response after receiving the SARS-CoV-2 mRNA vaccine (76): the antibody titer produced after receiving the first dose was over 10-times higher than that of kidney transplant recipients with no previous history of SARS-CoV-2 infection. These individuals experience an explosive increase in antibody titers during the second dose, which may be related to the immune memory triggered by previous infections. It is currently unclear how other organ transplant patients will respond to the vaccine, but the strategy of multiple vaccinations may be one of the effective measures to improve vaccine protection effectiveness. The primary organ affected by COVID-19 is the lung (77), and recipients of lung transplants are often at risk for breakthrough SARS-CoV-2 infections. Patients who underwent lung transplants and were administered Tixagevimab and Cilgavimab prophylactically had lower incidence of SARS-CoV-2 infection than those who were not (78). Some academics think that the creation of monoclonal antibodies that are effective against novel viral variations would be necessary for the successful implementation of the passive immunization concept (78).

In addition, SOTRs who have received the SARS-CoV-2 mRNA vaccine can still be infected with SARS-CoV-2. A UK study showed that, compared with SOTRs who had not received or only received one dose of SARS-CoV-2 mRNA vaccine, the mortality rate of SARS-CoV-2-positive patients who had received two doses of vaccine decreased from about 12 to 7.7% (79). Qin et al. studied breakthrough infections after receiving two doses of SARS-CoV-2 mRNA vaccine (80) and found that out of 18,215 SOTRs receiving two vaccine doses, 151 (0.83%) developed breakthrough infections, of whom 87 (58%) were hospitalized and 14 (9%) died. Compared to healthy individuals, SOTRs had an 82-fold greater risk of breakthrough infection and a 485-fold greater risk of hospitalization and death due to breakthrough infection. Wadei et al. (81) reported that some SOTRs have a low or undetectable immune response after receiving the SARS-CoV-2 mRNA vaccine. These patients developed breakthrough infections after receiving the first or second dose of the vaccine. Compared with all SOTRs receiving the SARS-CoV-2 mRNA vaccine in their center, the breakthrough infection rate in this population was 0.6%, significantly higher than the 0.05% breakthrough infection rate of all SOTRs. Overall, although vaccination with SARS-CoV-2 mRNA vaccine can effectively reduce the risk of SARS-CoV-2 infection in SOTRs, its level of protection is lower than that of the general healthy population. Therefore, while determining the safety of the vaccine, arranging for SOTRs to receive the SARS-CoV-2 vaccine as soon as possible can reduce the risk of omicron infection or reduce the likelihood of developing severe illness, maximizing safety for SOTRs and their transplant function.

## 6 Managing PLWH and transplant recipients in the omicron era and future directions

Many clinical studies have found that neutralizing antibodies targeting SARS-CoV-2 can not only be used to treat mild and moderate COVID-19 in patients with high-risk factors but also play an important role in pre- and post-exposure prevention of COVID-19. Univariate analyses of risk of hospitalization and death due to COVID-19 infection in secondary immunodeficiency have shown that neutralizing antibody replacement therapy, as a protective factor, can reduce the risk of hospitalization and death (82). In addition, some studies have shown that for patients with immune-mediated inflammatory diseases who receive B cell-depletion agents and vaccines, preventive use of tixagevimab/cilgavimab combined with active COVID-19 outpatient treatment before exposure may effectively reduce disease severity in this highly vulnerable population (83). Neutralizing antibodies have the advantages of good specificity, high safety, clear mechanism of action, easy mass production, and can be used for prevention and treatment at the same time. However, as inferred from the escape mutation spectrum, various single mutations in omicron SARS-CoV-2 can counteract neutralizing antibodies targeting different epitopes, and omicron may lead to significant immune escape and potential antigen transfer. It is crucial to analyze the reactions of different neutralizing antibodies to omicron, which will provide information for the development of drugs and vaccines based on these antibodies (84).

It is critical to acknowledge the review’s shortcomings. The COVID-19 study trend is changing quickly, which makes it challenging to reach definitive conclusions because clinical data will keep piling up quickly. Furthermore, the majority of the literature lacks clarity in identifying the many types of infected strains, and there are numerous possible sources of bias in all of these investigations. To ascertain the possible roles that could be performed in the topic, a thorough population-level analysis is required.

Overall, for HIV-infected individuals, active treatment should be given for the primary disease to adequately control the CD4^+^ count. For SOTRs, the adjustment of immunosuppressive drugs during COVID-19 infection needs to be cautious and individualized. Vaccination remains a legitimate and effective preventive measure for both groups of patients. Further research is required into the application of neutralizing antibodies in these populations, especially clarifying their role in immunosuppressed populations. Other aspects of omicron COVID-19, such as secondary infections, long COVID symptoms, and persistent active infection also require further research.

## Author contributions

YS: Investigation, Software, Writing – original draft. LL: Resources, Writing – original draft. KZ: Writing – review & editing.
